# A Time-Modulated Transparent Nonlinear Active Metasurface for Spatial Frequency Mixing

**DOI:** 10.3390/ma15030873

**Published:** 2022-01-24

**Authors:** Luyi Wang, Hongyu Shi, Gantao Peng, Jianjia Yi, Liang Dong, Anxue Zhang, Zhuo Xu

**Affiliations:** 1 School of Information and Communications Engineering, Xi’an Jiaotong University, Xi’an 710049, China; bigcrash@stu.xjtu.edu.cn (L.W.); gtpeng@stu.xjtu.edu.cn (G.P.); jianjia.yi@mail.xjtu.edu.cn (J.Y.); anxuezhang@xjtu.edu.cn (A.Z.); 2 MOE Key Laboratory for Multifunctional Materials and Structures, Xi’an Jiaotong University, Xi’an 710049, China; 3 Yunnan Observatories, Chinese Academy of Sciences, Kunming 650216, China; dongliang@ynao.ac.cn; 4 Electronic Materials Research Laboratory, Key Laboratory of the Ministry of Education, Xi’an Jiaotong University, Xi’an 710049, China; xuzhuo@xjtu.edu.cn

**Keywords:** nonlinear metasurface, time-modulated metasurface, varactor diode

## Abstract

In this article, a time-modulated transparent nonlinear active metasurface loaded with varactor diodes was proposed to realize spatial electromagnetic (EM) wave frequency mixing. The nonlinear transmission characteristic of the active metasurface was designed and measured under time-modulated biasing signals. The transmission phase can be continuously controlled across a full 360° range at 5 GHz when the bias voltage of the varactor diodes changes from 0 V to 25.5 V, while the transmission amplitude is between −2.1 dB to −2.7 dB. By applying the bias voltage in time-modulated sequences, frequency mixing can be achieved. Due to the nonlinearity of the transmission amplitude and transmission phase of the metasurface versus a time-modulated bias voltage, harmonics of the fundamental mode were observed using an upper triangle bias voltage. Furthermore, with a carefully designed bias voltage sequence, unwanted higher order harmonics were suppressed. The proposed theoretical results are validated with the measured results.

## 1. Introduction

The frequency mixer [1,2,3] is instrumental in a wireless communication system. By utilizing the nonlinear characteristic of components such as the diode or transistor [4,5], the electrical circuit produces new signals at the sum and difference of the original frequencies. Increasing types of frequency mixers such as passive mixers and active mixers are present in almost every wireless communication electronics system; however, the frequency mixer for a spatially propagating EM wave, with tantalizing applications in new architecture wireless communication systems [6,7,8], is more elusive.

As a type of surface EM component, a metasurface [9,10,11] with artificially designed structures exhibits novel properties for spatial EM wave manipulation, such as multibeam generation [12], beam steering [13], vortex beam generation [14] and so on. Furthermore, the EM response of the metasurface can be electrically adjusted by loading active devices on every unit cell [15,16,17,18], i.e., the smallest atom of a metasurface.

The digital coding principle [19] that links the metasurface with the digital world is also proposed. The basic idea is that the EM response of a metasurface, such as the reflection/transmission amplitude or phase can be digitalized (or discretized) and programmed to simplify the design, reduce the fabrication cost and, most importantly, to pave the way that links digital information with the coding metasurface. Researches on space-coding metasurfaces [20,21], temporal-coding metasurfaces [22,23] and space-temporal-coding metasurfaces [24,25] are being pursued by the science community and applications such as for EM scatter reduction [26], nonreciprocal effect [24], harmonic manipulation [22,23] and new architecture wireless communication systems [6,7,8] are proposed. In particular, temporal-coding metasurfaces that program the metasurface with time-modulated sequences show great possibility for manipulating harmonic distributions, i.e., to reshape the energy distributions of the incident wave between different harmonic orders.

Frequency mixing for a spatial EM wave, also referred to as harmonic manipulation, has been researched in [17,23,27,28,29,30] using the time-modulated metasurface. In those works, a reflection-type metasurface design with discrete EM responses is dominant; however, reflection-type designs inevitably suffer from blockages and are not the best option for system integration. Additionally, the discrete EM responses of the metasurface are realized using PIN diodes or switches, and although this simplifies the design and is beneficial for prototype realization, the discrete amplitude or phase response greatly impedes the frequency mixing quality since biasing sequences diversity is limited, resulting in high level unwanted higher order harmonics [23,27]. To summarize, a transmission-type metasurface with continuous EM responses that can be applied as a frequency mixer for a spatially propagating EM wave is still desired and a method to suppress unwanted higher order harmonics is also needed.

In this article, a transparent time-modulated metasurface with a continuous transmission phase modulation ability that functions as a frequency mixer of spatially propagating EM waves is proposed. The incident wave at a fundamental frequency of *f*_0_ can be up conversed to *f*_0_ + *f*_1_ when the varactor diodes in the metasurface is an applied upper triangular bias voltage signal with a period of 1/*f*_1_. In addition, benefitting from the continuous transmission phase modulation characteristic, unwanted higher order harmonics are further suppressed by applying a carefully designed nonlinear time-modulated bias voltage sequence.

## 2. Nonlinear Characteristic of the Active Metasurface

**Figure 1:**
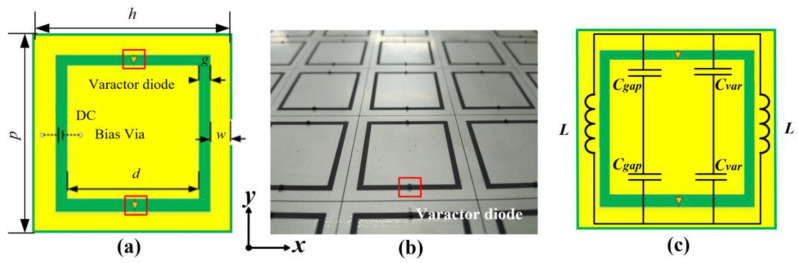
(**a**) Geometry of the unit cell. (**b**) The fabricated active metasurface sample. (**c**) Equivalent circuit of the unit cell.

An active metasurface which is capable of electrically tuning the transmitted EM wave phase [31] is utilized to analyze the frequency mixing of spatial electromagnetic waves. The unit cell of the active metasurface and the fabricated metasurface sample are displayed in Figure 1a,b. The unit cell is composed of a central metal patch and a metallic square loop printed on a substrate with a thickness of 1.524 mm, relative dielectric constant of 2.65, and a loss tangent of 0.0017. Two varactor diodes (SMV1405-079LF, Skyworks, Irvine, California, United States) are loaded between two metal areas for electrically tuning the capacitance between them. The geometric parameters are *p* = 33 mm, *h* = 32.8 mm, *d* = 24 mm, *g* = 1.5 mm and *w* = 3 mm. The unit cell design is evolved from a square slot FSS design [32] which exhibits a band-pass characteristic. When loaded with voltage-controlled-capacitance diodes, the unit cell can be equaled to an LC circuit as shown in Figure 1c, where for a normally incident wave (*y*-polarized), *L* represents the inductance of the metal patch, *C_gap_* represents the capacitance induced by the gap between the inner and outer metal patch, and *C_var_* represents the capacitance added by the varactor diodes. Therefore, it is possible to intuitively evaluate the relationship between the passband and unit cell parameters, which can be expressed by:

(1) $$f=\frac{1}{\pi \sqrt{L\cdot \left(C_{gap}+C_{var}\right)}}$$

The parameters of the unit cell are then simulated and optimized using CST Microwave Studio (Version 2016, Computer Simulation Technology GmbH, Darmstadt, Germany) to ensure good bandpass and transmission phase characteristics. Because of the limited capacitance varying range of the varactor diodes (0.79 to 2.01 pF in this design), the transmission phase modulation range for a single layer unit cell is low. This is solved by stacking multiple layers of the unit cells vertically, separated by an air gap [33]. Using a parameter study and experimental measurement, five layers of the stacked structure with a 3.5 mm air gap (approximately 16/λ for impedance matching) between each layer is sufficient to cover 360 degrees of the transmission phase while maintaining a good bandpass characteristic.

**Figure 2:**
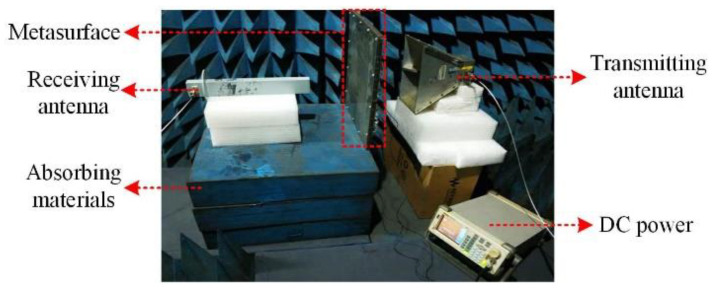
Diagram of transmission measurement of the active metasurface.

The fabricated metasurface was composed of 80 unit cells arranged in a square lattice. It is noteworthy that a margin of pure metal was designed to suppress the diffraction of the EM wave from the metasurface. The central unit cell was also covered with pure metal for vortex beam generation purposes as described in [31], which, however, posed negligible side-effects to the designed metasurface transmission characteristics. Five separate layers of the structure were stacked vertically with an air gap of 3.5 mm. Foams were inserted between the layers and nylon screws were distributed around the metasurface perimeter to enhance the stability of the metasurface. According to the experiment setup as illustrated in Figure 2, the transmission characteristic of the metasurface was measured from 4 GHz to 6 GHz by a vector network analyzer of Agilent E8363B (Agilent, Santa Clara, California, United States) in a microwave anechoic chamber. A direct current power amplifier PS-305 was used to supply all the varactors on the metasurface with a uniform bias voltage. The measured transmittance was collected, respectively, when the bias voltage increased with a step of 0.5 V from 0 V to 25.5 V.

**Figure 3:**
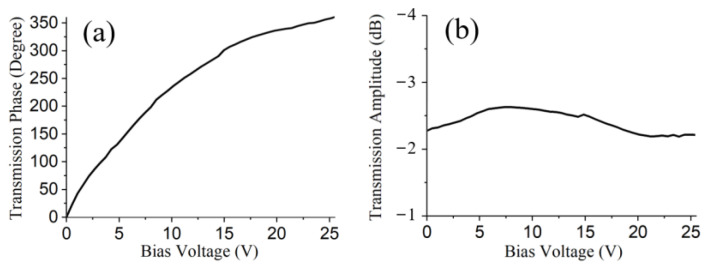
Transmission phase (**a**) and transmission amplitude (**b**) of the metasurface at 5 GHz versus bias voltage.

Then the transmission amplitude and phase at 5 GHz were selected from the measured data which is displayed in Figure 3. The transmission phase of the metasurface could be tuned from 0 to 360° with an increasing bias voltage, while the transmission amplitude was between −2.1 dB to −2.7 dB. The transmission loss was inevitably caused by the active varactor diodes on the metasurface which could be further alleviated by using low-loss active components.

After characterizing the transmission properties, we studied the case when a single-tone EM wave at the frequency *f*_0_ was incident on the metasurface and this is expressed as:

(2) $$y_{0}\left(t\right)=E_{0}e^{j2\pi f_{0}t}$$

If the metasurface is biased with a time-modulated voltage signal *v(t)*, the transmitted EM wave will be:

(3) $$y_{p}\left(t\right)=E_{0}\cdot E\left(v\left(t\right)\right)\cdot e^{j2\pi f_{0}t+jp\left(v\left(t\right)\right)}$$

where *p*(*v*) and *E*(*v*) are the nonlinear relationship of transmission phase and transmission amplitude versus the time-modulated bias voltage, respectively. If *v*(*t*) represents the upper triangle wave with a peak voltage of *V*_1_, as in:

(4) $$v\left(t\right)=\frac{V_{1}}{T_{1}}\cdot t,0<t<T_{1}$$

Then, through a Fourier transform, the spectrum of the transmitted EM wave can be expressed as:

(5) $$F\left(f\right)=a_{0}\delta \left(f-\left(f_{0}+f_{1}\right)\right)+a_{n}\delta \left(f-\left(f_{0}\pm nf_{1}\right)\right)$$

where *a*_0_ is the magnitude of the transmitted frequency-mixed EM wave and *a_n_* is the magnitude of the higher order harmonics at frequencies of *f*_0_ + *nf*_1_. Therefore, the frequency of the incident EM wave shifts up to *f*_0_ + *f*_1_, and the nonlinearity of the transmission phase and magnitude versus time-modulated bias voltage leads to higher order harmonics.

## 3. Results

**Figure 4:**
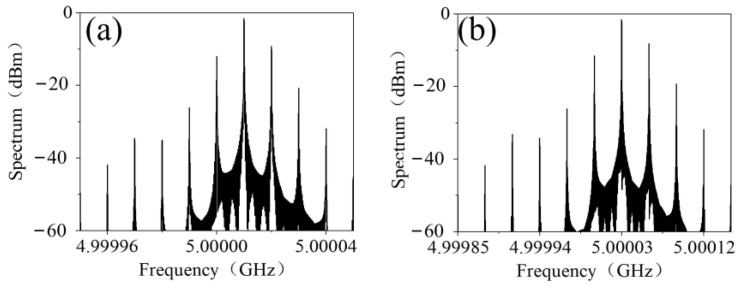
The simulated spectrum of the frequency-mixed transmitted EM wave biased with (**a**) 10 kHz and (**b**) 30 kHz upper triangle voltage signal.

The phenomenon of frequency mixing was numerically simulated using MATLAB (MathWorks, Natick, MA, USA). The bias-voltage-varying nonlinear transmission phase and amplitude of the metasurface was first obtained by fitting the measured data as illustrated in Figure 3 using cubic spline interpolation. Then the spectrum of the transmitted EM wave was calculated using a fast Fourier transformation. If the bias voltage was a time-modulated 10 kHz upper triangle signal, then the spectrum of the transmitted EM wave was as shown in Figure 4a. The central frequency of the transmitted EM wave shifted up to 5.10001 GHz and was 8 dB greater than the highest harmonics. Furthermore, as shown in Figure 4b, when the frequency of the time-modulated signal was up to 30 kHz, the central frequency of the transmitted EM wave shifted up to 5.10003 GHz with −6.8 dB maximum harmonics.

**Figure 5:**
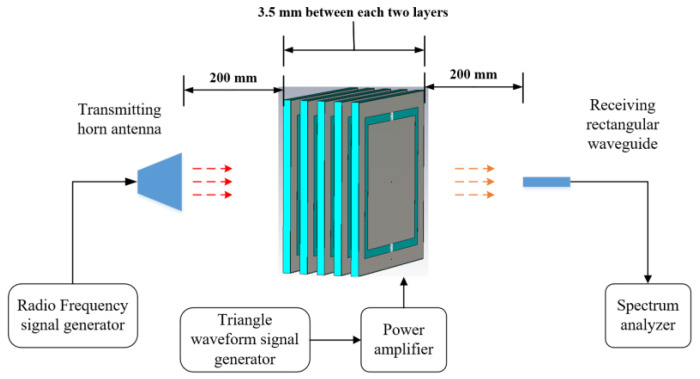
The block diagram of the measurement system.

Then the frequency-mixing measurement environment was set up, the block diagram of this is illustrated in Figure 5, which was basically the same as the setup in Figure 2 except for the additional triangle waveform generator. A horn antenna was used to radiate a single-tone 5.0 GHz EM wave onto the metasurface. The transmitted EM wave was received by a WR-229 open-ended rectangular waveguide probe (Hengda Microwave, Xi’an, Shaanxi, China) and its spectrum was acquired by a Rohde & Schwarz spectrum analyzer (Rohde & Schwarz, Muenchen, Germany). The distance between the metasurface to the transmitting horn antenna and the receiving rectangular waveguide were both 200 mm.

**Figure 6:**
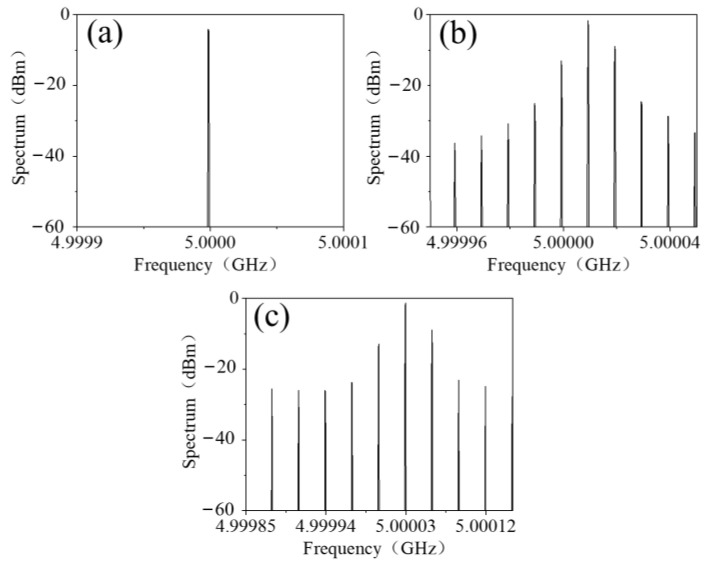
The measured spectrum of the frequency-mixed transmitted EM wave with (**a**) no voltage biasing biased and with (**b**) 10 kHz or (**c**) 30 kHz upper triangle voltage signal.

Firstly, no bias voltage was applied to the varactor diodes loaded on the metasurface and the spectrum of the transmitted EM wave exhibited a single-tone 5 GHz as displayed in Figure 6a, indicating that no frequency mixing occurred. Then, the bias signal was set as a 10 kHz upper triangle signal and was further amplified to a maximum of 25.5 V by a high-speed bipolar amplifier. As shown in Figure 6b, the amplitude maximum of the spectrum was at 5.10001 GHz and was 7.3 dB greater than the other higher order harmonics, indicating that the fixing mixing of the spatial EM waves was up to 5.10001 GHz. It is worth explaining that the measured spectrum was sampled at a discrete frequency with an interval of 10 kHz, as opposed to the simulated results; however, the spectrum distribution between the interval was negligible and did not affect the measured results. Similarly, when the bias signal was set as a 30 kHz upper triangle signal, the central frequency of the transmitted EM wave shifted up to 5.10003 GHz as shown in Figure 6c with −7.6 dB maximum harmonics.

Due to the intrinsic nonlinear transmission versus the time-modulated bias voltage of the proposed metasurface, harmonics of a higher order would appear, which may have tainted the frequency mixed spectrum, further posing a challenge to possible applications such as for direct modulation communication [6,7,8,34,35]. In order to reduce the interference of higher order harmonics, the method of harmonics suppression using a carefully designed biasing voltage waveform is proposed.

Let *v*(*p*) be the inverse function of *p*(*v*), and a nonlinear bias voltage *v_b_*(*t*) is as follows:

(6) $$v_{b}\left(t\right)=v\left(\frac{2\pi }{T_{1}}t\right),0<t\leq T_{1}$$

Then the transmission phase will be:

(7) $$p\left(t\right)=p\left[v_{b}\left(t\right)\right]=p\left[v\left(2\pi f_{1}t\right)\right]=2\pi f_{1}t$$

where *f*_1_ is the period of bias voltage. Thus, the transmitted EM wave can be rewritten as follows:

(8) $$y_{p}\left(t\right)=E_{0}\cdot E\left(v\left(t\right)\right)\cdot e^{j2\pi \left(f_{0}+f_{1}\right)t}$$

Compared with Equation (2), the higher order harmonic components caused by nonlinear *p*(*v*) are eliminated.

**Figure 7:**
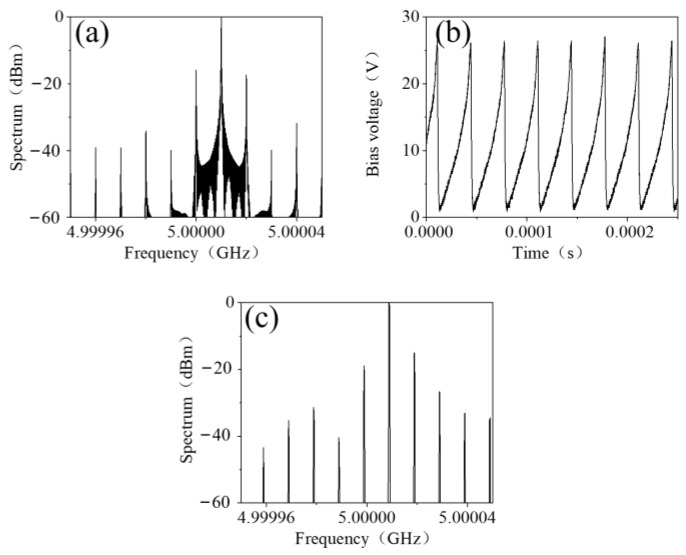
(**a**) The simulated spectrum of the frequency-mixed transmitted EM wave biased with a 10 kHz voltage waveform *v_b_*(*t*), (**b**) a 10 kHz voltage waveform *v_b_*(*t*) and (**c**) the measured spectrum of the frequency-mixed transmitted EM wave biased with a 10 kHz voltage waveform *v_b_*(*t*).

In order to observe the effect of the harmonics’ suppression, the spectrum of the transmitted EM wave was simulated when the bias voltage signal was *v_b_*(*t*) with the frequency *f*_1_ of 10 kHz. As shown in Figure 7a, the central frequency shifted up to 5.10001 GHz and the harmonic reduced to −16 dB. Then the waveform of *v_b_*(*t*) was imported to an arbitrary waveform generator RIGOL 3101A and was amplified to 25.5 V, as shown in Figure 7b. The spectrum of the transmitted EM wave was measured and shown in Figure 7c. The central frequency shifted up by 10 kHz and the highest harmonics were −14.1 dB, which is in good agreement with the theory and the simulation result.

Finally, a table of comparisons between our work and other frequency mixing methods is presented in Table 1. From the comparison results we can infer that the literature on metasurface-based spatial frequency mixing schemes are centered on a reflection-type design with a harmonic suppression level ranging from 0.2 dB to 10 dB. Ref. [17] is singular as it realized a second harmonic generation (SHG) using a lumped frequency multiplier chip, and by virtue of the performance of the active chip, a low harmonic level was realized. To summarize, our transmission-type, continuously modulated metasurface-based spatial frequency mixer with unwanted harmonics suppression, offers a novel solution to manipulating the spatial wave.

## 4. Conclusions

In conclusion, we proposed a transmission-type metasurface capable of continuously controlling the transmission phase of the incident EM waves across 360 degrees at 5 GHz. The proposed transparent metasurface, functioning as a frequency mixer for spatially propagating EM waves was characterized and validated. The theoretical simulation and experimental measurement were in good agreement with each other, showing that by applying a triangle wave signal with a frequency *f*_1_ (10 kHz) to the varactor diodes loaded on the metasurface, the central frequency (5 GHz) of the transmitted EM wave will shift by *f*_1_, indicating the frequency mixing characteristic of the proposed scheme. Furthermore, the proposed nonlinear bias voltage sequence was analyzed and unwanted higher order harmonic waves were suppressed. Compared with the schemes in the literature, our transmission-type design avoids the blockage effect induced by a reflection scheme and a continuous modulation ability enables a nonlinear bias voltage sequence that further suppresses unwanted higher order harmonics. The transparent active metasurface and the proposed biasing schemes offer a route to realize spatial EM wave manipulation.

## Figures and Tables

**Table 1:** Comparison of harmonic manipulation schemes in the literature.

Ref	Reflection or Transmission Type	Discrete or Continuous Modulation	Harmonic Suppression Level (dB)
[17]	Reflection	N/A	30
[23]	Reflection	Discrete	0.2
[27]	Reflection	Discrete	8
[28]	Reflection	Continuous	2
[29]	Reflection	Continuous	10
[30]	Reflection	Discrete	10
**Our Work**	**Transmission**	**Continuous**	**14.1**

## Data Availability

The data presented in this study are openly available.
